# G-DaM: A Distributed Data Storage with Blockchain Framework for Management of Groundwater Quality Data

**DOI:** 10.3390/s22228725

**Published:** 2022-11-11

**Authors:** Sukrutha L. T. Vangipuram, Saraju P. Mohanty, Elias Kougianos, Chittaranjan Ray

**Affiliations:** 1 Department of Computer Science and Engineering, University of North Texas, Denton, TX 76203, USA; 2 Department of Electrical Engineering, University of North Texas, Denton, TX 76203, USA; 3 Department of Civil and Environmental Engineering, University of Nebraska-Lincoln, Lincoln, NE 68588, USA

**Keywords:** smart agriculture, Internet of Agricultural Things (IoAT), Blockchain (BC), Distributed Data Storage (DDS), edge system, groundwater quality data management

## Abstract

Groundwater overuse in different domains will eventually lead to global freshwater scarcity. To meet the anticipated demands, many governments worldwide are employing innovative and traditional techniques for forecasting groundwater availability by conducting research and studies. One challenging step for this type of study is collecting groundwater data from different sites and securely sending it to the nearby edges without exposure to hacking and data tampering. In the current paper, we send raw data formats from the Internet of Things to the Distributed Data Storage (DDS) and Blockchain (BC) edges. We use a distributed and decentralized architecture to store the statistics, perform double hashing, and implement access control through smart contracts. This work demonstrates a modern and innovative approach combining DDS and BC technologies to overcome traditional data sharing, and centralized storage, while addressing blockchain limitations. We have shown performance improvements with increased data quality and integrity.

## 1. Introduction

Water acts as an essential supporting element of life. In total, 96% of the water resides in oceans, and the remaining 3% of freshwater comes from sources such as rain, streams, rivers, lakes, and groundwater. About 1.69% of the freshwater comes from the ground [1] and is used mainly for agriculture and industry, which has put more pressure on global water resources. As the population is predicted to grow in the coming decades, so is the increased demand for food and crop yields. Groundwater utilization has expanded rapidly through water withdrawals and central pivots for irrigation and domestic purposes. Our higher dependency on water will result in the reduction in groundwater and its availability for the dependent life systems. Soil absorbs rainwater to store water in the ground [1]; however, due to global warming, rainfall patterns have been changing, affecting the sinking amount of water and gradually decreasing the earth’s freshwater supply. Similarly, using fertilizers excessively may increase nitrate contamination due to leaching, and a possible reduction in groundwater availability [2,3].

Data are the primary driving force for science. Data for groundwater availability are collected from different sources, such as an aquifer, climate science, law, public policy, and hydro-geology, with the help of sensors. The sensors for collecting agricultural data for fields are referred to as part of the Internet of Agricultural Things (IoAT). IoAT devices collect the statistics with suitable sensors in their raw format to recognize the problems. The devices collect unlimited data 24/7, which is helpful for later analysis. However, the IoAT is useful for collecting data, but it comes with its constraints that are discussed in more depth in Section 2. Research and study on multiple data contexts received from these IoAT devices are complicated; combining and integrating all of these into a single platform is a more difficult challenge. Food production can increase with unlimited water resources; hence, data collection on agricultural farms is crucial. The entities involved in sharing the knowledge and technology from the groundwater sectors are minimal, which raises new issues from a political point of view. The data collected help researchers to construct different visualization, simulation, and study models to analyze groundwater reserves and calculate water levels for the next generation. Although data gathering helps in a significant way, incorrect information can lead to misleading analyses. Researchers and experts are more worried about the authenticity of the data because they may have been tampered with and modified in the data path [4]. Using the blockchain is one possible solution for researchers to avoid data integrity and quality problems.

**Figure 1:**
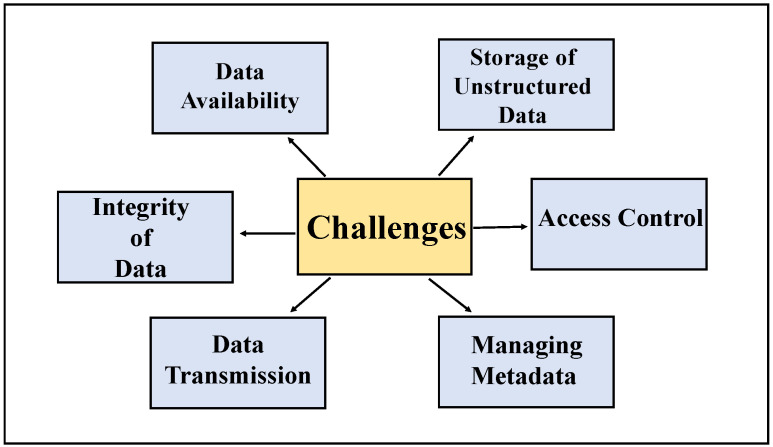
Groundwater data management challenges.

Storage systems with a central design face issues such as Internet dependency risks in data confidentiality, single-point failures, latency problems, and security, and are more prone to data attacks. Information gathered from different sources comes in various formats that need to be brought under one mode for sharing and storing. Some of the challenges included in managing groundwater data are listed in Figure 1. Advanced technologies such as the blockchain and distributed data storage methods can provide several benefits to overcome the issues encountered.

The blockchain delivers a decentralized architecture that uses cryptographic hashes for security to create immutable blocks comprising data transactions ordered in chain blocks. These chains of blocks are equal in size and have timestamps embedded. To validate the data transactions and secure them from malicious attacks, the blockchain uses complex mining protocols [5]. Smart contracts execute logic and act as small services for application program interfaces to implement access control. Although blockchain is famous for its immutable data transfer, it could be perfect for such applications. High fees, massive energy requirements, and slow data validation during increased traffic are a few of its challenges. Therefore, we perform distributed data storage with the help of an Interplanetary File System (IPFS). Progress in employing these technologies is taking place in different fields such as smart agriculture [6] and intelligent medical things [7] to ensure greater security for sensitive data. This paper highlights blockchain’s and DDS’s plausible role in supporting groundwater data management.

The current paper is presented in the following order. By combining and extracting meaningful information from different fields of the groundwater discipline, we establish the present work. In Section 2, the problems with the current groundwater data management systems are discussed along with solutions. Prior related work and sources for groundwater data are discussed in Section 3 and Section 4, respectively. A novel architecture for the proposed G-DaM and algorithms are presented in Section 5, and Section 6, respectively. The implementation of the system is detailed in Section 7 followed by the validation of the system in Section 8. Finally, Section 9 presents the conclusions for the current paper, and also outlines future research.

## 2. Novel Contributions

### 2.1. Problem Definition

In conventional data storage systems, latency issues, IoT limitations, higher mining times, time-bound storage, and higher transaction costs are some of the main problems that can arise. We introduce an intermediate edge embedded with DDS and blockchain technologies to take in more extensive data, avoid central issues and maintain privacy and immutability when sharing the groundwater records. We use an interplanetary file system for DDS and the Ethereum public blockchain in the current application to overcome all the above challenges. Next, we discuss some of the problems and itemized novel solutions.

### 2.2. Current IoAT Challenges

**Figure 2:**
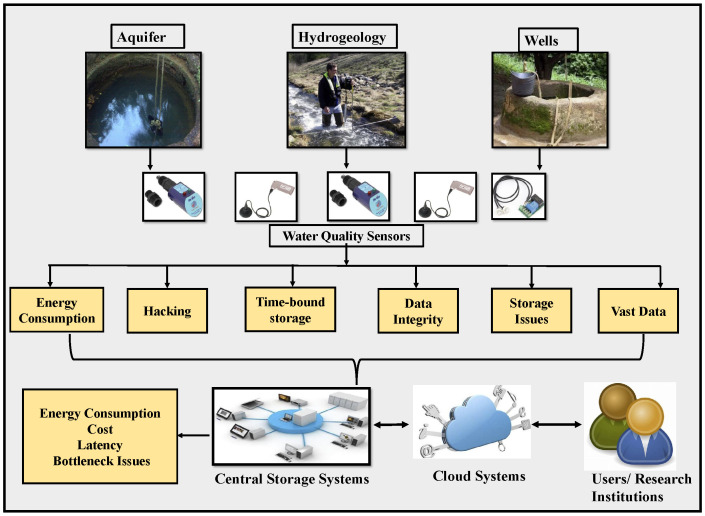
Current IoAT, cloud and central system challenges.

Agro-things work extensively without pause 24/7 to collect groundwater data, consuming high energy. The data collected are vast, and if they are not sent for storage in databases, more statistics can be lost due to time-bound storage limitations, which could have been helpful for research. Most current agro-things use central and cloud systems for storage. If the data in a centralized model lead to incorrect statistics, there is a possibility that every other device connected can be corrupted. During data transmission, these things can lose data integrity, trust, and quality as they can be hacked and tampered with easily. Figure 2 shows the challenges that occur in IoAT, cloud, and central systems used in Smart Agriculture for groundwater data collection. IoAT machines cannot process data securely and can increase latency issues using traditional methods for storage. However, IoAT devices, cloud, and central storage systems have undergone improvement in terms of distributed storage systems and studies implementing energy-efficient strategies have been performed [8,9,10]. Our current work tries to implement distributed methods to overcome these issues.

### 2.3. Importance of Data Quality in Groundwater Data Transmission

Data that are accurate and of a high quality play an essential role in forecasting the threats and dangers that can help in avoiding future disasters for humanity. Contamination of groundwater is a severe threat, and a global issue which can be can be caused by chemicals, road salt, bacteria, viruses, medications, fertilizers, and fuel. Wrong data predictions of groundwater quality can lead to dangerous health hazards, degrade the quality of the environment and impact socioeconomic development. A discussion of real-time disasters that have occurred due to groundwater contamination to show the importance of quality data transmissions is available in [11]. People staying near the river Woburn in Massachusetts in 1969–1979 were affected due to river pollution with industrial solvents. There have been traces of high water contamination which causes various diseases, including leukemia, liver, kidney, prostate, and urinary cancer. To overcome the water crisis in the city of Flint, the pipeline has been shifted to the river of Flint from the Detroit River and Lake Huron. Due to the high content of lead and other contaminants in the drinking water, many health problems, such as skin lesions, hair loss, high lead levels in the blood, vision loss, memory loss, depression, and anxiety, were observed in the people. In New Delhi, most water pipelines are connected to the Yamuna river. It is a highly contaminated river, and the reasons for its contamination include pesticides, copper, zinc, and nickel, due to which people are facing health issues such as death, disease, cancer, and organ damage.

### 2.4. Why Blockchain in Data Transmission?

With blockchain, data transmissions can be performed with increased trust and quality. The communication between the entities or the stakeholders between the data collecting fields and the end systems can be achieved more securely and authentically using the blockchain because it acts as a ledger system. Once we write data on the blockchain, it cannot be reverted or tampered with as it uses encryption techniques to calculate a hash of the data transmitted. Using this property as an advantage in securing the relevant statistics makes blockchain suitable for sharing the data. Data storage in blockchain uses a decentralized architecture to hinder centralized storage issues. Although it has many benefits in securing the information gathered, it is more costly to store data with blockchain because of the gas (mining) fees it requires for each transaction. The advantage of a decentralized architecture is that it will not have a severe effect if a single node fails because other nodes will continue to function. Through this, it maintains adequate redundancy within the network. The data gathered are distributed among nodes and encrypted so only the owner can view the data. The blockchain takes care of data using the following two techniques: sharding and swarming. Sharding allows the file to be divided into smaller chunks for a quicker transfer. Some percentage of the node is retained for sharding in each transaction. The participants do not receive the entire file; instead, they are sent a part of the file. Only the owner knows the locations of the shards through a private key which is also beneficial when discovering shards. Swarming is a technique that keeps all the shards together and helps in decreasing latency while retrieving the files from the nearest nodes [5].

### 2.5. Past Incidents of Insecure Data in Water Plants

In February 2021, the water treatment plant in Oldsmar, Florida, was attacked by a group of hackers who were able to gain access to the operations technology system. The attack mainly aimed to increase the sodium hydroxide content in the water from 100 parts per million to 11,100 parts per million. That attempt was prevented by an operator who stopped the attack by reversing the toxic levels in the water [12]. A hacker attempted to poison a water plant in San Francisco Bay Area in January 2021. The hacker had all the details of a former employee’s TeamViewer account with which he could delete all the programs required for water plant treatment [12].

### 2.6. Problem Addressed in the Current Paper

- Groundwater data management challenges can be classified into storage, pre-processing, and secure sharing. Attributes such as integrity, availability, security, access, ingestion, metadata, transformation, and warehousing can be sub-categorical. Figure 1 illustrates different kinds of data management issues.
- Central storage vulnerabilities.
- Disadvantages of the blockchain for slow speed, energy-draining, scaling, and price.

### 2.7. Solutions Proposed in the Current Paper

- DDS through IPFS for off-chain storage to evade blockchain limitations.
- A blockchain-based data storage solution to overcome IoAT challenges.
- Access control approaches through blockchain smart contracts.
- Achieving privacy by combining both DDS and blockchain technologies.

### 2.8. State-of-the-Art Solutions

- For improving the quality, overcoming IoAT constraints, and decreasing the uncertainty of the data, unique blockchain technology is used for groundwater data sharing and storing.
- For bulk data to be stored and shared, DDS is used, providing increased security to the derived statistics.
- A state-of-the-art architecture is presented for the current G-DaM with dual hashing security included.
- A result log is shown for comparing transaction times, fees, and costs between traditional blockchain and blockchain with distributed storage systems.

## 3. Prior Related Works

Water quality data are collected using different platforms. The information gathered in these applications plays an essential role for water managers and researchers in making correct decisions and further analyses. The system in [13] is designed with different modules to gather water quality and query data with statistical charts using a client–server architecture. It sends collected reports through traditional central systems. The study in [14] employs GIS (geographic information systems) for the management of water quality information. The data are interpreted and collected in the form of geographic data and stored in traditional database tables and spatial records. In recognizing the quality and quantity of the water in aqua agriculture, the approach in [15] is implemented using a big data platform built on the SpringBoot and JPA frameworks and a traditional database for storing and sharing the data among farmers. Others [16] use Autonomous Surface Vessels (ASVs) for capturing data in shorter time periods with lowered costs. The data are stored either by utilizing the ASV onboard software, which is not efficient for real-time visualization, or traditional central servers. The pH level is measured for getting water quality in the domestic supply [17]. The sensor gives information regarding the water’s quality and the tank’s water level near residential areas. The data collected are sent to cloud systems and to mobile users for alerting purposes. The application in [18] mainly concentrates on the security of the data gathered through the Internet of Things using blockchain at every level, i.e., from the device layer to the communication level. Real-time water quality data are collected in [19] to detect any violation records using blockchain and to ensure privacy and integrity in the data flow.

With the help of an information system and centralized techniques, a client–server architecture with a single database sector is developed in [20]. As the groundwater data are stored in different geographical divisions, the paper introduces a single system for a more straightforward and accessible analysis. Other visualizations and analysis techniques are performed in [21] to compare two-dimensional and three-dimensional images with the help of fuzzy queries and relational databases. The database is used for storing important WebGIS water information that is collected from diverse sources. The storage for different groundwater data formats in [22] is completed using a distributed framework. The structure makes use of ArcIMS Services for spatial metadata handling. All the metadata management is achieved through central systems with the help of the RDF/XML platform and the J2EE environment. By using the web-based central system in [23], the groundwater data are composed and managed. It proposes a unified framework for collecting, storing, and sharing over a vast network of data workers and end-system users.

While these methods for monitoring and managing water quality data increased the information quality and achieved a united structure, limitations still need to be addressed in the power usage, cost, computation, and access control areas. Some are solely designed using a single blockchain, increasing the cost and energy consumed, while others practice web services and are dependent on centralized servers for storage. Ref. [24] discusses the limitations of traditional data sharing, centralized storage, and blockchain more elaborately, along with a study on how the blockchain is helpful in mitigating these problems. Relying on the cloud for data processing is risky because the system can have a single point of failure and unknown accesses. As there is an increase in groundwater utilization, it is necessary to verify its availability for future generations. Accurate studies need to be performed based on the facts collected, so we utilize distributed storage strategies with blockchain for access control and integrity. As groundwater data are one of the most critical forms of data, authenticity and access permissions are required for sharing the data among stakeholders. Blockchain is an efficient way to share data when dealing with sensitive information. Its functionality is similar to an immutable ledger that keeps a log of every transaction in sequential order. The consensus mechanism in the blockchain further provides immutability, permanency, and anonymity to the groundwater records. It mitigates different threats such as tampering, repudiation, disclosure of the information, and denial of service, which need to be fulfilled for a higher quality of groundwater data. DDS supports storage in a decentralized way using peer-to-peer network models that share the file across different nodes or computers. The file is broken into smaller parts and distributed among a network of end systems to track the file with hashes. Table 1 presents different domains and data management strategies developed for information administration using diverse platforms and technologies. To the best of our knowledge, the current design combining DDS and Blockchain security is the first such attempt at groundwater data management.

## 4. Sources for Groundwater Data

**Figure 3:**
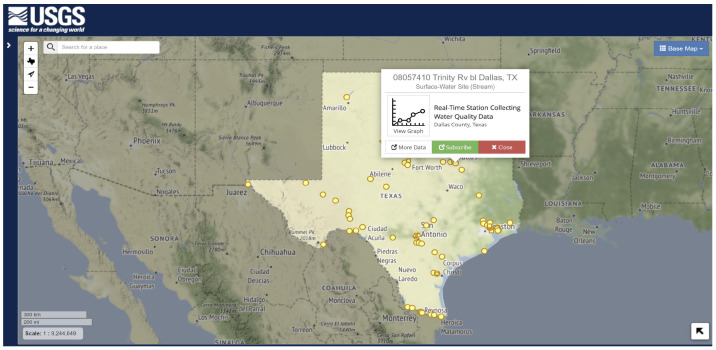
Water Quality Data Collection Sites of USGS -Texas.

**Figure 4:**
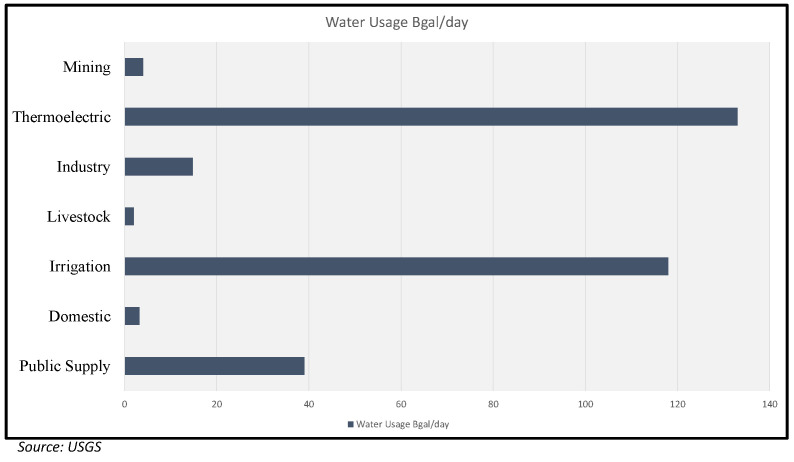
Groundwater and Water Quality Data Users.

The data can be collected using different techniques and platforms, such as remote sensing, multimedia, spatial, and other sources. The information gathered for nitrogen content in crops [25] is in a geospatial format, which differs from data in text or numerical formats. For securing and storing each of these types of data, experts use different methods. Figure 3 shows the available sites set up by the United States Geological Survey (USGS) for collecting water quality data in the state of Texas. These data-collecting centers record water quality and send the information to nearby institutes for making decisions and further research. For data scientists to suggest solutions, they must fully comprehend the water quality statistics and data origin. The U.S. Geological survey conducted in 2015 shows the water usage, which can be seen in Figure 4 [26]. The information gathered can be broadly categorized into structured and unstructured. The data in the structured format are in a table form, also called a relational database. In contrast, unstructured data include video, audio, text, and images that require a complicated structural design for sharing and storing.

### 4.1. Activities on Field

One of the primary sources of data are observations collected during field operations. The activities include drilling, pumping, and monitoring operations. The information gathered with these techniques is robust in terms of accuracy. Drilling and pumping operations tend to be occasional, while monitoring is performed quarterly or less frequently [27]. This type of data collection is structured and typically performed locally within an aquifer; although, the recent addition of sensors allows for off-site data collection.

### 4.2. Historical

Historical data are in an unstructured format and contain legacy reports, physical maps, and text documents. Digitizing and transforming these sources of information into machine-readable data can create a new stream of more critical data [28].

### 4.3. Remote Sensing

This type of source forms data using primarily satellite, airborne, or ground-based instruments for observations [29]. They contain both structured and unstructured formats that are multi-dimensional, heterogeneous, and have increasingly voluminous datasets.

### 4.4. Computer Simulation

Hydrological data are generated through computer models that use numeric methods and simulation techniques. Atmospheric models and land surface models apply complex mathematical equations to predict weather forecasts and integrate hydrological data with biological and radiation-based processes on land [30]. The source contains both structured and unstructured formats with multi-dimensional, heterogeneous, extensive data.

### 4.5. Web and Social Media

With the emergence of the Internet, a new way of communication and transfer of information is practiced. Web and media can include text, images, videos, or audio, forming an unstructured data format [31]. Mostly, this source type is found on web pages and social media posts.

### 4.6. Internet of Things (IoT)

Connected devices are intelligent equipment that can join each other and digital systems over the Internet. These “things” continually stream environmental statistics. IoT systems can generate and collect large amounts of data faster than conventional or manual data collection techniques. With increasing demands to make applications smart, intelligent things are also growing. IoT fields include city, home, agriculture, medical, and industrial fields. Smart agriculture is a field that involves different IoT Sensors to collect data on humidity, water range, light, etc. [32]. They gather information and connect to the farmer using mobile devices to detect farming field conditions remotely. Some of the smart developments are briefly discussed here to show their relevance. Ref. [33] presents a unique device for crop disease predictions, irrigation, and crop selection in an automatic method with a solar sensor node. It can also capture crop images with continuous sensing. Another innovative agricultural application [34] is a clever greenhouse to increase yield and adapt to farming changes with changing environments. With the help of smart IoT devices, medical statistics are also collected, where control sharing and access management are essential. With added blockchain immutability in [35], a smart pillow-Internet of Medical Things (IoMT) application is built for stress control and supervision.

### 4.7. Groundwater and Groundwater Quality Data User Domains

**Figure 5:**
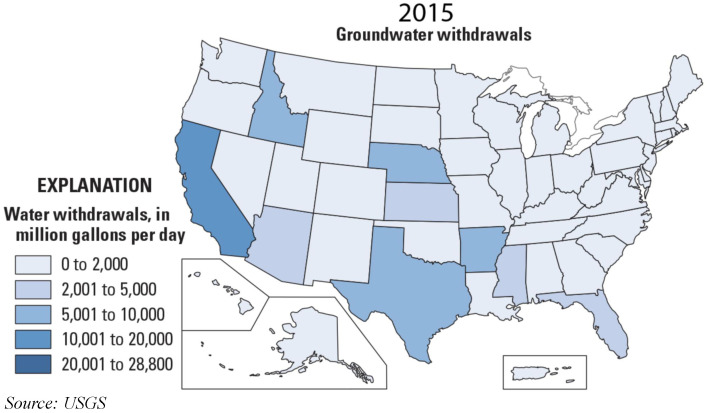
Groundwater withdrawals in United States.

Here, we discuss the receivers of the groundwater and the actors that benefit from the high quality groundwater data [36]. Private and public distributors distribute the water supply to the public through withdrawals and connect them to parks, swimming pools, fire departments, and wastewater treatments. These water supplies also include water distribution for residential and domestic needs for drinking, sprinkling, and washing. The agricultural division for growing fruits and vegetables to supply food for the world population is the most crucial recipient of groundwater and its quality data. The groundwater used in irrigation should be free from chemicals to obtain healthy produce. Livestock is another area that requires high levels of groundwater and quality data. The animals on the field require water for drinking, sanitation, and other hygienic purposes. Thermoelectric power is generated by sending water to turbines that circulate between heat exchangers to produce electricity. A huge percentage of water is also sent to industrial use for manufacturing daily usage products and is also essential for controlling the dust during the mining process. All these sectors utilize water as their primary source. Figure 5 shows the groundwater withdrawals across the United States.

## 5. A DDS and Blockchain Platform Water-Quality Data Management System Architecture

Measuring water quality is required as more groundwater is becoming contaminated through its overuse, storage tanks, pollution, septic tanks, uncontrolled harmful waste, and medical waste in drinking water supplies. Sensors are used to collect data and send them to end systems for sharing and storing. Different sources discussed in Section 4 are helpful in gathering and storing the information from their respective end stations. These end systems can also be referred to as edge system nodes that need to provide data integrity, privacy, storage, and security while transmitting the data. Each of these nodes participates by combining DDS storage and blockchain functionalities to create a unified and orchestrated method to manage groundwater data.

### 5.1. Interplanetary File System (IPFS)—DDS

In Section 1, we discussed some of the limitations of blockchain for validating and storing large amounts of data; with this constraint, off-chain storage for information is a feasible solution. Deciding which information stays on-chain and which goes off-chain is essential. Storj1, FileCoin2, Sia3, and IPFS are some off-chain storage examples. Data can be kept secure using off-chain methods to distribute the files among various nodes using encryption and shredding techniques.

The IPFS decentralized file-sharing platform recognizes the documents and folders through content. It mainly depends on the distributed Hash table (DHT) to recover the locations of the file and information regarding node connectivity. When a file gets uploaded to IPFS from the end station, it is divided into 256 KiloByte maximum length segments. IPFS blocks are referred to as segments to differentiate blockchain blocks from IPFS blocks [37]. Every segment is recognized using a cryptographic hash calculated according to its content, called a content identifier (CI). A Merkle-directed acyclic graph (Merkle DAG) depicts a complete file through its root hash and can be used to rebuild a file from its segments inside the IPFS.

A DHT works on the principle of a distributed key-value store. It uses distance metrics along with node identifiers to store and reclaim the information quickly. When reading for the value, the end systems try to find other nodes close to the key and obtain the value/content. To write a value, the nodes establish already defined end stations that are most relative to the key and inform these nodes of the key attribute value, using buckets inside the network to track nodes [38].

IPFS makes use of S/Kademlia [39] for DHT. This secured Kademlia algorithm provides two distinct forms of information. Firstly, when a file is uploaded from the end station, this node registers itself as a file segment provider. Secondly, DHT provides information regarding how to connect to the node with the help of an identifier. In this way, the IPFS node appeals to the providers from DHT and links to retrieve a file.

### 5.2. BC-Ethereum Smart Contract

Ethereum is one of the popular blockchain application development tools. Transactions in Ethereum are completed using a cryptocurrency called ether, and smart contracts are used to write the main application logic. The solidity programming language is used to design the contract, and when it compiles, a bytecode is generated that is understandable only by the Ethereum Virtual Machine (EVM). Smart contracts are mainly Turing complete and can be utilized for various purposes. Ethereum primarily works in a decentralized way that ensures that the control for executing is not in the hands of nodes and embeds trust using a consensus mechanism. With this trusted method, data in the transactions cannot be changed or modified. The access control procedures such as variables, mappings, and structures can be used in the solidity programming language and called using conditional statements. If these statements meet the norms, the state is not modified; if they don’t, the state returns to its original value.

Inside the smart code, a state variable can be coined to assign a value to store on the blockchain. An owner state variable can be called inside the contract migrations and assigned to the msg.sender(). The variable’s value is defined inside the constructor function and called on whenever the smart contract is created for the first time or deployed to the blockchain. As solidity is a statically typed language, we can declare a variable as the string datatype and enable the public to access the value outside of the contract [40]. For writing and reading the values inside the state variable, the programming language provides functions such as set() and get() along with multiple access control functions such as amIOwner(), amIOwnerMultiple(), checkAccess(), and checkAccessMultiple(). To make Ethereum’s states persistent, we can declare them constant.

### 5.3. Architecture

**Figure 6:**
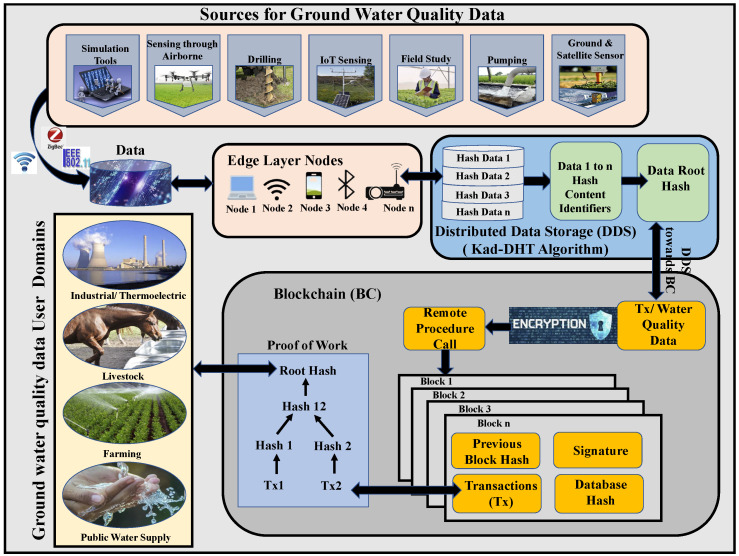
Proposed Blockchain Architecture for Groundwater Data Management with DDS.

A setup of the DDS-IPFS platform is developed between the data source and the blockchain to communicate with the smart contract inside the blockchain. It acts as a mediator for moving the transactions to the methods of smart contracts for taking control of the storage and communicating with the network gateways and DHTs. The currently proposed system G-DaM architecture is given in Figure 6. Here, the data traveling from the IPFS to the blockchain are represented as transactions.

#### 5.3.1. Adding File

When the end system submits a groundwater data file, the IPFS creates segments of the file with a corresponding Merkle DAG and content identifiers and provides the hash string as the output. The secured Kademlia protocol consists of subprotocols to identify and verify the node through Content Identifiers. Some nodes may be unreachable due to network address translators and firewalls; IPFS overcomes these nodes through filtering. Each object in IPFS storage includes two fields, one for the data and the other for links. The data field contains binary data, which are of a specific size. The links field is further divided into the link name, a hash of the linked object, and the linked object size. Every node or peer that has IPFS as the form of distributed storage maintains a routing table with links for other peers. A routing table decides where the moving data should be inside the network.

#### 5.3.2. Linking IPFS Data to Ethereum Smart Contracts

There are two types of accounts in Ethereum, namely externally owned accounts and contract accounts. With the help of private keys, Ethereum addresses, and digital signatures, the externally owned accounts can hold the ether cryptocurrency to perform transactions. The same follows with contract accounts, but the difference is that they are controlled through programming code. Private keys are at the core of the Ethereum accounts, and they determine the Ethereum address, referred to as the account. Access control and monitoring of the data are achieved through digital signatures created using private keys. To be included, the transaction inside the blockchain Ethereum transactions requires a valid digital signature. Any peer who obtains the private key can become the transaction owner; therefore, keys are stored in particular files and Ethereum wallet software such as metamask. Ethereum makes use of public-key cryptography.

Registering the hash string file from IPFS inside the smart contract is carried out using addBlock functions, and the transactions are verified based on the CI’s. The calling set() function inside the contract writes the hash string file as a transaction to the block. Elliptic curve cryptography (ECC) multiplication is applied to the transaction data. ECC is a one-way function where the multiplication is performed in a single direction but is impractical to reverse. The private key owner can create public keys and share them with different nodes, realizing that no node calculates the function to obtain the private key. This arithmetic method provides secure digital signatures which make the transaction data tamper-resistant with total ownership and control of the contracts. The transactions are listed as a Merkle binary hash tree which can help to add new blocks to the previous chain. The protocol produces hashes in a bottom-up direction and avoids fake groundwater files from the beginning through a proof of work (PoW) consensus mechanism. The root hash on the tree acts as the digital footprint to make the transaction block valid. The PoW algorithm confirms transactions or the data in the blocks and adds them to the chain. This algorithm mainly uses mathematical puzzles that can be solved. Those who solve them are miners, and the process is mining. Once the hash string from IPFS is valid and added to the blockchain, it generates a transaction hash on the blockchain explorer etherscan to retrieve the file.

#### 5.3.3. Retrieving the File

Inside the smart contract, the get() function is defined and called to read the file whenever requested by the owner or nodes with the correct permissions. Once the required authorizations are provided, a groundwater user sector node can request and obtain the corresponding files. To achieve this, the user node checks for the transaction hash content identifier with the source checksum content identifier to retrieve and reassemble the file. If there are no authorizations provided in the contract, there is no reply to the request.

## 6. Algorithms for DDS and Blockchain Based Framework

From the edge systems (E_d_S), the data move towards the IPFS, and from there to the blockchain, as stated in Algorithm 1. Public-key cryptography and SHA-256 are used in distributed data storage for hashing the uploaded files. Both private and public keys are generated, respectively, for each edge system to control access, to provide unique messages called digital signatures and for signing the groundwater quality data file. The file uploaded to the edge system is given as F_L_. The react JS used for the front-end design oversees the file uploaded. Once the water quality data file is submitted, it is converted into the buffer (E_d_S), B_uf_ file of each 256 kB B_uf265 KB_. The buffer file is attached with the private key and is then signed. The IPFS digitally signs the hash string/hash message “h(B_uf_)” produced; and h denotes the hash function. The signed hash string is then called by the set() function in the smart contract. With the help of the elliptic curve digital signature algorithm (ecdsa), a signature output of the “h(B_uf_)” is generated. To order the Ethereum objects, an encoding technique called recursive length prefix (rlp) is used. p_k_ represents the signing private-key of the blockchain, and e is the RLP encoded data. F_un keccak256_, F_un signature_ represent the functions for the keccak-256 hash and signing algorithm, respectively. Once the data are hashed/signed twice, the smart contracts help in reading and writing the transaction for the blockchain using access rules.
**Algorithm 1** Data from Groundwater endsystems to IPFS and blockchain.  1:E_d_S, BC generate their respective Public and Private Keys (P_u_E_d_S, P_r_E_d_S) and (P_u_BC, P_r_BC)  2:E_d_S(FL)→B_uf_→B_uf265 KB_.  3:S_C_[set()]→B_uf_265 KB→DDS.  4:The file gets hashed through cryptography method using SHA 256 to give distinct fingerprints represented as C_I_(Content Identifiers).  5:P_u_E_d_S = h(P_r_E_d_S * C), where C acts as a constant, * is a mathematical operation that is calculated in single direction and H is the secured hash function.  6:**if** FL==h(P_r_E_d_S * C)==h(B_uf265 KB_) **then**  7: Publishing h(B_uf265 KB_)→DDS, using IPFS client.  8: S_C_[get()] and S_C_[Publish()] functions to publish “h(B_uf265 KB_)” from DDS.  9: Signing “h(B_uf265 KB_)” with esdsa, Signature = F_un signature_ (F_un keccaK256_(e),p_k_k).10: Attaching the ecdsa signature to the transaction.11: **if** “h(B_uf265 KB_)” is signed with ecdsa algorithm **then**12:     The hash maps in S_c_ are used for accessing the IPFS hash string towards ethereum accounts.13:     Hash map has device owners, address and device id as key along with with hash string encrypted that is written on Blockchain.14:     The write access policy checks for the validity of the data and functions in S_c_ help is publishing the encrypted data.15:     **if** Device owner and address are related device id. **then**16:         Runs the Write operation.17:     **else**18:         Deletes Write operation.19:     **else**20:         Process End.21:     **else**22:         Process End.23:     **end if**24:    **end if**25:**end if**26:Repeat the steps from 1 through 26 every time edge system collects groundwater quality data.

The steps for recovering the data from the blockchain to the user domains (U_d_) are provided in Algorithm 2. The user domains should have the signature values and ordered transactions for retrieving the file. In the water quality data signed, private and public keys for creating the signatures are also present. The user domain ensures the water quality data are signed to authorize the signature and check if the hash functions have been compromised. Only the user domains with appropriate values can contact and receive the file. A complexity of O(1) [39] is required for validating and solving the cryptographic puzzles.
**Algorithm 2** Data from Blockchain to User Domains.  1:BC and U_d_ generate their respective Public and Private Keys (P_u_BC, P_r_BC) and (P_u_U_d_, P_r_U_d_).  2:The requester sends for data access request.  3:The access request gets signed by Requester’s private key (P_r_A_r_) and the signature gets attached along with data request.  4:The request for data access is concatenated with the signature an is then encrypted by public key of Edge system (P_u_E_d_S) for publishing from the client side Smart contract.  5:The request gets decrypted by the Edge System and uses signature for verifying the data integrity.  6:**if** Signature matches **then**  7:    The permission for reading the data is given to the requester.  8:    The owner, address and the id details of the device are provided by the requester.  9:    The owner, address, and id of the device are maintained in the smart contract hash map along with the registered user domains.10:    **if** owner, address and id of requester matches hash map of smart contract **then**11:     data can be accessed to read by the requester.12:    **else**13:     Declined the data access.14:    **else**15:     Process End.16:    **end if**17:**end if**18:Repeat the steps from 2 through 18 every time there is a new user sector access request.

## 7. G-DaM Implementation

**Figure 7:**
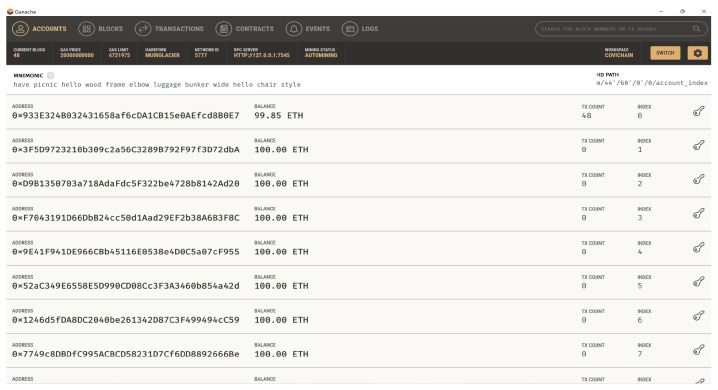
Ganache local blockchain.

**Figure 8:**
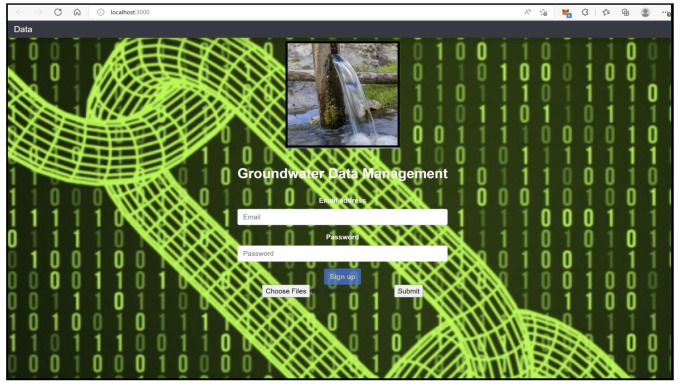
G-DaM User Interface.

Some dependencies are significant for the DDS application design, which are briefly discussed here. Ganache is a personal blockchain platform that is mainly used for deploying smart contracts, application development, and running tests locally that mirror actual public blockchain. Figure 7 shows ten free accounts provided by the mirror blockchain Ganache for developing distributed applications. Ganache initiates by setting up a platform for writing smart contracts with the help of a nodes package manager (Npm) and truffle framework (Tf). The local nodes are initiated with Npm, and Tf provides different tools for developing the present application. The tools in Tf help with smart contract management, testing in an automated way, contract migration and deployment, network management, running scripts for JS client code, and developing client-side code [41]. For the front-end design of the application, the react-java script (reactJS) framework is used, as shown in Figure 8.

The Infura IPFS gateway has an ipfs-http-client package that can be installed using a local node. The package can be called from the front-end reactJS for attaining distributed storage for the current G-DaM application. Another essential package that is used for communicating Ethereum and local nodes is web3.js. The front end of the G-Dam system is connected to the backend blockchain by configuring the Tf to the Ganache host address 127.0.0.1:7545. A regular browser cannot be used for communicating with the blockchain; instead, a metamask extension browser is helpful. The metamask also handles personal accounts, funds, and fees for data transactions. The logic code inside the smart contract helps in interacting with the string data generated from IPFS which are forwarded to the blockchain.

Testing is one of the crucial stages of application development. Blockchain testing plays a vital role since contract code execution on an actual blockchain will lead to higher risks due to its non-reverting property. The G-Dam application here is tested using Tf in local Ganache to verify its efficiency and deployed in the Ropsten test network for live setting performance testing without the use of real ether and mainnet tokens.

## 8. G-DaM Results

**Figure 9:**
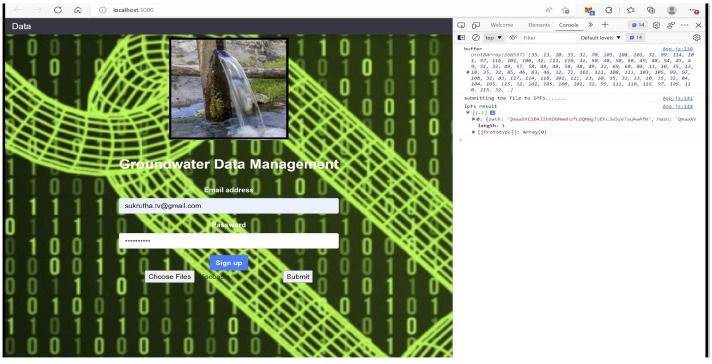
File to buffer to hash.

We submit the water quality data file to the front-end to read the input in the form of a buffer, and the resulting IPFS hash string is delivered, as shown in Figure 9.

**Figure 10:**
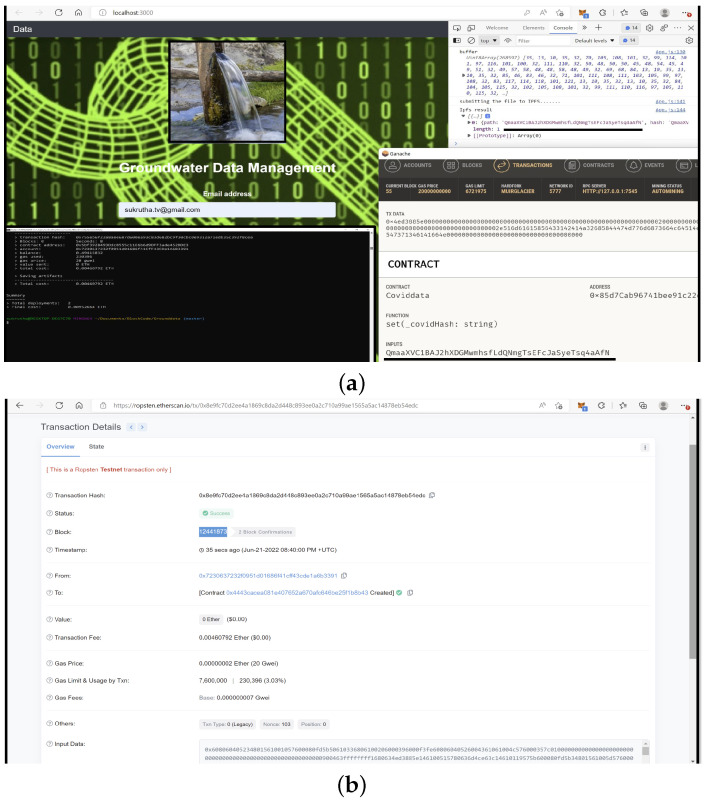

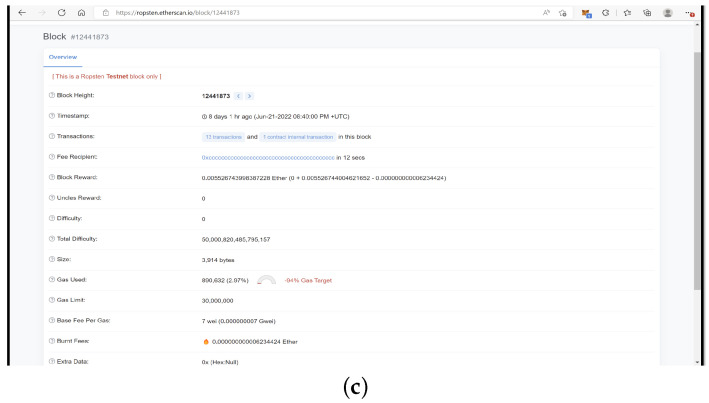
Dataflow from User Interface to Back-End Blockchain. (**a**) Hash verification & deploying to ropsten. (**b**) Transaction/Blockchain Hash. (**c**) Transaction Validating Time.

The metamask ethereum wallet acts as a connection medium between the user interface and Ganache. The hash string is generated from the front-end form linked to DDS-ipfs. Once the hash is received, the metamask asks to confirm the transaction to store the ipfs hash on the blockchain, which in turn provides a cryptographic transaction hash. Both the ipfs hash string output and the Ganache input are verified to be the same, as underlined in Figure 10a, and then deployed to ropsten testnet, which mirrors the functionality of the actual mainnet. Once deployed to the testnet, the transaction hash is provided along with the status, timestamp, block number, ether used, and the gas used, as shown in Figure 10b,c. The complete flow of data for the current G-DaM application is shown in Figure 10.

### Datasets

The datasets we used for testing the current application are given in Table 2. These datasets comprise the water quality data for each state in the United States and are collected from the US Geological survey [42]. The datasets are initially compressed into a .zip format. We tested each data sample for its integrity, privacy, quality, and security through double hashing, one executed with ipfs and the other with the blockchain, as given in Table 3.

**Figure 11:**
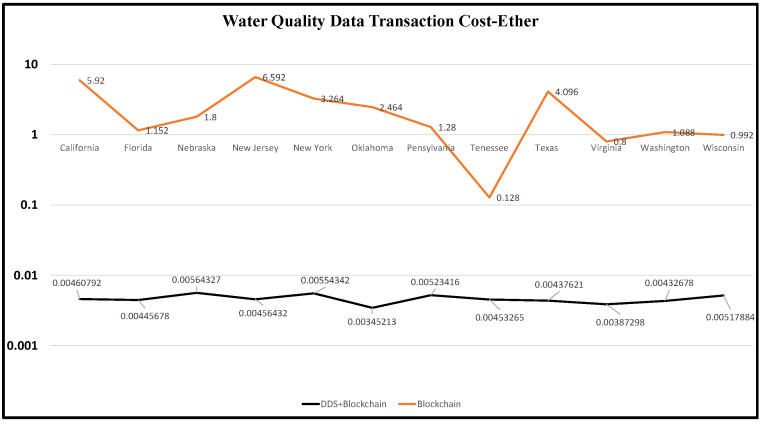
Comparing Tx-Cost for water quality data flow between blockchain-only and blockchain with DDS.

The information regarding one ether(eth) price is $1098.84, and the mining time is 13.96 s for 1 MB of data [43] as of 30 June 2022. For 1 KB of data to be shared and stored on the blockchain, it would require 0.032 ether fees [43]. Based on these facts, we calculated the transaction costs for all our water quality datasets and compared the prices between blockchain and blockchain with DDS, as shown in Figure 11.

## 9. Conclusions and Future Direction for Research

This paper provides a state-of-the-art design combining DDS and blockchain for the management of groundwater quality data. It solves various issues of central system challenges, blockchain latency, data integrity problems, privacy, and data quality issues. The blockchain uses ECC cryptographic puzzles on the data hashes received from the DDS, which acts as a form of extra protection for groundwater quality data. The DDS s/kademlia protocol avoids churn, eclipse, and Sybil attacks by inducing strong cryptographic signatures and hashing procedures. This paper also proposes a novel architecture and platform for stakeholders in groundwater quality data management and helps initialize digital agreements. For the control of access and data, the current paper makes use of public blockchain smart contracts. With the help of a private blockchain, the present application can be made more confidential and will have increased control over the quality of data flow.

## Figures and Tables

**Table 1:** Data management and storage approaches for water Quality.

Application	Data Storage	Security Level	Cost	Computation
Urban Rural Water Quality Data [13]	Centralized	Low-High Risks on Data	High	High
Water Quality Data with GIS [14]	Centralized	Low-High Risks on Data	High	High
Water Quality information in Big data [15]	Centralized	Low-High Risks on Data	High	High
Water Quality data with ASV [16]	Centralized	Low-High Risks on Data	High	High
Water Quality Data from IoT [17]	Centralized	Low-High Risks on Data	High	High
Water Quality Data from IoT [18]	Decentralized	High-Single Hashing	High	High
Water Quality Data from IoT [19]	Decentralized	High-Single Hashing	High	High
Groundwater quality Data [20]	Centralized	Low-High Risks on Data	High	High
Groundwater quality Data [21]	Centralized	Low-High Risks on Data	High	High
Groundwater quality Data [22]	Centralized	Low-High Risks on Data	High	High
Groundwater quality Data [23]	Centralized	Low-High Risks on Data	High	High
**G-DaM [Current-Paper]**	**Decentralized-OffChain storage**	**High-DoubleHashing**	**Low**	**Low**

**Table 2:** Datasets for G-DaM.

Data Name	Dataset Size	Compressed.zip Size	Link
California Water Quality	1.64 MB	186 KB	https://waterdata.usgs.gov/ca/nwis/qw (accessed on 10 October 2022)
Florida Water Quality	328 KB	36 KB	https://waterdata.usgs.gov/fl/nwis/qw (accessed on 10 October 2022)
Nebraska Water Quality	709 KB	84 KB	https://waterdata.usgs.gov/ne/nwis/qw (accessed on 10 October 2022)
New Jersey Water Quality	1.76 MB	206 KB	https://waterdata.usgs.gov/nj/nwis/qw (accessed on 10 October 2022)
New York Water Quality	883 KB	102 KB	https://waterdata.usgs.gov/ny/nwis/qw (accessed on 10 October 2022)
Oklahoma Water Quality	669 KB	77 KB	https://waterdata.usgs.gov/ok/nwis/qw (accessed on 10 October 2022)
Pennsylvania Water Quality	385 KB	40 KB	https://waterdata.usgs.gov/pa/nwis/qw (accessed on 10 October 2022)
Tennessee Water Quality	20 KB	4 KB	https://waterdata.usgs.gov/tn/nwis/qw (accessed on 10 October 2022)
Texas Water Quality	1.12 MB	128 KB	https://waterdata.usgs.gov/tx/nwis/qw (accessed on 10 October 2022)
Virginia Water Quality	191 KB	25 KB	https://waterdata.usgs.gov/va/nwis/qw (accessed on 10 October 2022)
Washington Water Quality	288 KB	34 KB	https://waterdata.usgs.gov/wa/nwis/qw (accessed on 10 October 2022)
Wisconsin Water Quality	262 KB	31 KB	https://waterdata.usgs.gov/wi/nwis/qw (accessed on 10 October 2022)

**Table 3:** Water quality Data sharing with double hash refuge.

File	File-Size	IPFS-Hash	Tx Hash/BC Hash	Tx Deploying Time (s)
California Water Quality data	186 KB	QmcMnYyywy5No 5eP25gcRirPymv4YAFL s3AyamC66X6dpv	0x9c9ff748384e2 3a50ddfcc6f2fbca49 ce55638e1b6136e 51d50bed19fb60b37c	8
Florida Water Quality data	36 KB	QmTTSJLxoAYSgQFpA q5z2MmSMuq1NfMY6 MGogKoSVbMhgw	0x833374419e5ac21 9f7f3591df7335ad508d0 bd6865897da3a935 212662fd051d	8
Nebraska Water Quality data	84 KB	QmY3y84FBmnzc2 EukKS3wyT6J5teGnT 3Y5aMXKhfGAW65C	0x3e65d503b14aed 2bbc1e4c393da861 857f1b137c9f185322 dec77c6cb41dea84	32
New Jersey Water Quality data	206 KB	QmSkQ2FsCywsfkv EiFmQwWY97evqWk CBqBgEBUNpLZd1tE	0x82e3011ea9c91 0d76a2faf759310920 3378a6950c3c2e8d8 2dbd2ebc29bed5fc	20
New York Water Quality data	102 KB	QmYmKPhKWvGs7 R1guBnPpwk8usNXqn 7j4ikX1ByvKtUagh	0x71285afe6a050cde bdd4c2e650cca2d3759 8ab459e3a0a77c5 19b1b87bbecc54	36
Oklahoma Water Quality data	77 KB	QmeDzZvmzkkCgf mC8UN8NbVT18oavX 7ZEtTVmpsirj4ndu	0x7ab98459b29b5 71fb654dbf90f884167dc4 4c8386115c381d8c9e 3c831611853	8
Pennsylvania Water Quality data	40 KB	QmPDXu4qMJHQR MTJC2T3rCB9CfFzQhRD thW6HsbRLUogo2	0xfdd3de4eb8b3 3d82120df40187fb51 b1fe6d4bcd1074df0519 80e6c5e5233210	20
Tennessee Water Quality data	4 KB	QmU4BmcNbTb uTe9LQxkTSHPiWmN9xj3F 9uQu624sieQVGs	0x8e9fc70d2ee4a 1869c8da2d448c89 3ee0a2c710a99ae156 5a5ac14878eb54edc	32
Texas Water Quality data	128 KB	QmVoN2iNU3T zDPy1QrG8Ck2nHMrqt PcAZN72E4i1MtPKsf	0xc9360e9e1d5b7d6 be2c8d9811ca427407 82aaf10c6a72866813b d4484c26c20d	20
Virginia Water Quality data	25 KB	QmRZDbew3iU9U gH3S9WZhPgi2n4gAq nUR7uvd9v67cncfD	0x9d547180ce0b f1f437f3f3934c1f759 bbfdbab8fc47c22c 73903e8f46392cb6f	8
Washington Water Quality data	34 KB	QmT5GrgoPH92nu a5WTbCUcDpiCs2RWC kxVkqJnRY7CY3Jq	0xf86cd670ff4e6 74f522d64badf7b 2674ac9a3846bbd91 b863f8ed012f944317	8
Wisconsin Water Quality data	31 KB	QmYTPr445A72L uscbaavgqppZKmMKrAY 9HV3U7dmbBB5dF	0xc544ef6ded8dc 865ada99b79b74faeae f897a55bc4c827c21 1fa9da95f758b68	20
